# Relative Contribution of Nitrogen Absorption, Remobilization, and Partitioning to the Ear During Grain Filling in Chinese Winter Wheat

**DOI:** 10.3389/fpls.2018.01351

**Published:** 2018-09-19

**Authors:** Bangwei Zhou, Maria Dolores Serret, Jordi Bort Pie, Syed Sadaqat Shah, Zhijian Li

**Affiliations:** ^1^Key Laboratory of Vegetation Ecology, Ministry of Education, Institute of Grassland Science, Northeast Normal University, Changchun, China; ^2^Unit of Plant Physiology, Faculty of Biology, University of Barcelona, Barcelona, Spain

**Keywords:** nitrogen remobilization, nitrogen accumulation, Rubisco, ear, wheat

## Abstract

Knowledge of the function of the ear as a key organ in the uptake, remobilization and partitioning of nitrogen is essential for understanding its contribution to grain filling and thus guiding future breeding strategies. In this work, four Chinese winter wheat genotypes were grown on a ^15^N-enriched nutrient solution. N absorption and further remobilization to the flag leaf, the ear and the mature grains were calculated via the ^15^N atom % excess. The results indicated that the high yields of the Chinese wheat genotype were determined by higher grain numbers per ear, with greater plant height and a larger ear size, while the thousand-grain weight did not affect grain yield. In the mature grains, 66.7% of total N was remobilized from the pre-anthesis accumulation in the biomass, while the remaining 33.3% was derived from the N taken up during post-anthesis. From anthesis to 2 weeks after the anthesis stage, the flag leaf remobilized 3.67 mg of N outwards and the ear remobilized 3.87 mg of N inwards from the pre-anthesis accumulation in each plant. The positive correlation between ear N_rem_ and grain N_rem_ indicated that the ear was an important organ for providing N to the grain, whereas the remobilized N stream from the leaves was not correlated with grain N_rem_, thus indicating that flag leaf N was not translocated directly to the grain. The grain N_rem_ was negatively correlated with the ear N concentration throughout grain filling, which suggested that higher-yielding genotypes had better sink activity in the ear, while Rubisco played a critical role in N deposition. Therefore, to improve yield potential in wheat, the N accumulation in the ear and the subsequent remobilization of that stored N to the grains should be considered. N accumulation and remobilization in the ear may at least be valuable for Chinese breeding programs that aim at optimizing the sink/source balance to improve grain filling.

## Introduction

In order to maintain yields the amount of nitrogen (N) fertilizer has been increased dramatically in recent decades and this trend will continue. This is particularly the case for wheat where many developing countries now rely on large quantities of chemical fertilizers (Foulkes et al., 2009). For example, in the case of China, most of the breeding has been conducted under conditions of no N-limitation (Zhou et al., 2014) and the cultivars released recently push farmers to rely on the over-use of chemical N fertilizer to maintain the yield potential. However, meeting future food security needs for the growing population does necessarily require a further increase in the amount of N fertilizer. In order to reduce the use of chemical N fertilizer and avoid environmental pollution while maintaining acceptable grain yields, breeding of highly N-efficient cultivars has become one major goal in recent plant breeding programs in China (Zhao et al., 2006). In summary, although a dramatic increase in genetic gains has been achieved in recent wheat breeding programs in China (Zheng et al., 2011; Xiao et al., 2012), the traits conferring greater efficiency in the use of N are not yet fully understood. Grain N is mainly sustained by: (1) the amount of N accumulated in the plant before anthesis, (2) the N taken up from the soil after anthesis, and (3) the N balance and redistribution among organs during grain filling (Masclaux-Daubresse et al., 2010; Gaju et al., 2014). It has been traditionally and widely reported that the major source of N required for filling the grains is accumulated in the shoots and roots during vegetative growth stages (Pang et al., 2014). In a study conducted using ^15^N labeling on a duplex soil at East Beverley, Western Australia, the contribution of pre-anthesis N to the grain was 2.2, 3.7, and 5.1 g m^−2^ when 15, 30, and 60 kg N ha^−1^ were applied, respectively (Palta and Fillery, 1995) and these contributions accounted for 60, 82 and 95% of the total grain N. In another experiment conducted by measuring the differential organ N content under field conditions (Arduini et al., 2006), the contribution of N remobilization to N grain content varied (from 40 to 60%) among genotypes but was not affected by plant density.

It is reasonable to consider that the genotypes with high N remobilization efficiency (NRE), where N is stored, would help to satisfy the need for better grain quality (Barraclough et al., 2014). However, the ability to correlate genetic variation in modern Chinese wheat varieties with the partitioning of N to different plant parts or even with the identity of the plant organ that contributes to grain N is substantially missing from the literature (Zhou et al., 2016). Leaf blades (and to a lesser extent the sheaths) were classically considered to play the most important role as sources of N remobilization due to their high protein content (Bidinger et al., 1977; Araus et al., 1986; Lopes et al., 2006). This is because at least half of the soluble protein in photosynthetic plant organs is comprised of Ribulose Bisphosphate Carboxylase Oxygenase (Rubisco) (Parry et al., 2003), with N being recycled following protein hydrolysis and export in the form of amino acids to grains during grain filling (Aranjuelo et al., 2013a; Zhou et al., 2016). However, a deeper understanding of processes conditioning N assimilation and remobilization in different organs is needed. In particular, the role of the ear as a source of N for filling grains has been rarely emphasized (Sanchez-Bragado et al., 2017), in spite of the fact that recent reports have shown that the ear may play a critical role as a photosynthetic contributor providing carbohydrates during grain filling (Aranjuelo et al., 2013b; Sanchez-Bragado et al., 2014a, 2016).

To study the contribution of the ear in terms of N accumulation and remobilization to filling grains is not straightforward due to the complexity of genotype by environment interactions (Barbottin et al., 2005). The specific quantification of the N available at the whole shoot level as well as for each specific organ, not to mention the assessment of N dynamics at the plant and organ levels (from N plant uptake to its further accumulation in the different photosynthetic organs and the subsequent remobilization to the growing grains), are still limiting the methodologies of evaluation. However, ^15^N isotope labeling is considered as a viable method for evaluating N partitioning and remobilization, and is less biased and more precise than the usual “apparent remobilization method” (which calculates the difference between the amount of total N present between anthesis and harvesting in different plant parts) (Kichey et al., 2007; Masclaux-Daubresse et al., 2010; Sanchez-Bragado et al., 2017). As described in a ^15^N labeling study conducted under field conditions (Kichey et al., 2007), on average 71.2% of grain N originates from remobilization, with significant differences due to genotype, and the other 28.8% is derived from absorption, with the flag leaf exhibiting higher N remobilization efficiency (NRE, 0.76) than the stem (0.73) and the ear (0.73). NRE depends on the amount of N remobilized to the grain in the post-anthesis period and on the amount of N stored in vegetative parts at anthesis (Gaju et al., 2014). However, many of the studies investigating NRE have been conducted at a coarse level that evaluated remobilization from “straw” (Palta and Fillery, 1993; Barraclough et al., 2014). In that context, elucidating the existence of genotypic variability in NRE among modern Chinese cultivars, particularly how N taken up by the plant is partitioned to individual organs and subsequently remobilized to grain, still needs to be understood. This is particularly important because recent breeding in China has placed emphasis on selecting genotypes with a larger sink-size, which means to breeding for bigger ear size or greater grain number per ear. Nevertheless, the question about the remobilization capacity of organs of modern Chinese wheat cultivars, particularly those adapted to the highly fertile provinces of central China surrounding the Yellow River (such as Henan and Shandong) (Zhou et al., 2007), remains unsolved.

The aim of this investigation was to determine the rate of N absorption and remobilization of the ear and the flag leaf blade, and their contribution to the N accumulated in grain for recently released high-yielding Chinese winter wheat varieties. Four cultivars were grown under natural environmental conditions. N uptake by the plant and the relative accumulation and further remobilization to grains was assessed through ^15^N labeling and estimation of N allocation among organs. The Rubisco content was quantified to assess to what extent the changes in the N content of the ear and the flag leaf during grain filling were caused by Rubisco synthesis and degradation.

## Materials and methods

### Experimental design

The experiment was performed outdoors in the natural environmental condition in the Experimental Field Facilities of the Faculty of Biology, University of Barcelona (Barcelona, Spain), from Nov. 4th 2016 to June 8th 2017. Four Chinese bread wheat (*Triticum aestivum* L.) cultivars were evaluated; Yumai 2, Lankao 198, Yumai 20, and Zhoumai 18, which have been released and mainly distributed in the Henan Province of China. The cultivars used in this experiment were selected based on their similar phonologies and different yield performances, which have been described in a previous field experiment (Zhou et al., 2014, 2015). The genotypes were planted in 30 L polyvinyl chloride pots filled with sand. Plant seeds were vernalized in a cold chamber for 14 days before planting. Twenty seeds were planted in each pot (the pot area was 0.05 m^2^) to simulate 400 seeds per square meter. These four genotypes were planted in a total of 24 pots (4 genotypes × 3 blocks × 2 pots for each plot = 24 pots) in a layout as a randomized complete block design with 3 blocks. From sowing to anthesis the seedlings were drip irrigated with a whole-strength Hoagland solution in all the pots. Between the 2 pots per plot, one was labeled with ^15^N while the other one was designated as the ^15^N background treatment (natural abundance ^15^N treatment). At anthesis, the substrate of one pot in each plot was washed with abundant water to remove the normal N in the substrate. One pot from each plot was labeled from anthesis until maturity with ^15^N-enriched Hoagland solution. In this treatment, the KNO_3_ was replaced by 10% ^15^N-enriched K^15^NO_3_ to chase a 0.5% labeling percentage (^15^N/total N). The other pot of each plot continued being irrigated with normal Hoagland solution as the ^15^N background for calculating the ^15^N atom excess. During the whole growth period, each pot was irrigated with approximately 50 L of Hoagland solution. The accumulated precipitation was 243.3 mm from 1 January to 8 June, and the most concentrated rainfall occurred in March and June. The environmental parameters were recorded at different phenological stages. During booting, heading, anthesis, and the milk stage, the mean ambient temperatures were 13.2, 15.5, 14.2, and 17.0°C, respectively, while the integrated solar irradiation levels were 16.6 MJm^2^, 22.2 MJm^2^, 22.0 MJm^2^, and 21.0 MJm^2^, respectively. During plant growth, the lowest temperature was −1°C on the 18th of January, while the highest temperature was 29°C on the 3rd of June.

### Plant sampling and harvesting

When all the genotypes reached anthesis and before the substrates were washed to remove the natural ^15^N, 3 plants were harvested from each pot, with the flag leaves and the ears separated and plunged into liquid N prior to storage at −80°C for later use. Another 3 plants were harvested from each pot, with the flag leaves and ears separated and oven dried at 60°C and then ground to a fine powder. After 2 weeks of ^15^N labeling, a similar number of plants as previously collected at anthesis were sampled and were also preserved as frozen and dry samples, respectively. Chlorophyll content was estimated at anthesis and 2 weeks after, in the middle of flag leaf blade from 5 different plants, using a portable meter (Minolta SPAD 502 Meter, Plainfield, IL, United States). Before harvesting, plant height was measured from the ground to the top of the awns. At maturity, all the plants from each pot were harvested, threshed to release grains and oven dried until they reached a constant weight. Grain number per ear, ear number per plant and thousand grain weight were determined from a set of 5 plants per plot.

### Stable nitrogen isotope signature and N concentration in total organic matter

The stable nitrogen (^15^N/^14^N) isotope ratios and N concentration were analyzed in the flag leaves and ears samples of both labeled and non-labeled plants at anthesis and 2 weeks after anthesis, as well as in mature grains. Flag leaf, ear and grain samples (about 0.7 mg of fine powder each) were placed into tin capsules for analytical determinations. The measurements were analyzed using an elemental analyzer (EA1108, Series 1, Carlo Erba Instrumentazione, Milan, Italy) coupled with an isotope ratio mass spectrometer (Delta C, Finnigan, Mat. Bremen, Germany) in a continuous flow mode at the Scientific Facilities of the University of Barcelona (Spain). For the ears and flag leaves, the N quantity was expressed relative to total organic matter unit. The amount of ^15^N atoms in labeling samples was calculated by atom% abundances (*A*) as described by Robinson (2001): *A* = ^15^N/(^15^N + ^14^N) ^*^ 100, where ^15^N and ^14^N are the numbers of ^15^N and ^14^N atoms present in the plant sample, respectively. In this work, atom% ^15^N excess was calculated as the difference between the *A* of labeled plant samples and the corresponding *A* of non-labeled plants from the same plot. IAEA N_1_ and IAEA N_2_ ammonium sulfate and IAEA NO_3_ potassium nitrate were used as secondary isotope standards of known ^15^N/^14^N ratios for calibration to a precision of 0.2‰.

### Assessment of N remobilization and uptake in organs

For each ear and flag leaf, the quantity of N originating either from post-flowering absorption (N_abs_) or from remobilization of the N accumulated during pre-anthesis (N_rem_) was estimated according to Kichey et al. (2007) by using the following equations:

(1) $$\begin{array}{l} N_{abs}=\frac{\left[N_{final}*\left(E_{final}-E_{flo}\right)\right]-\left[N_{flo}*\left(E_{flo}-E_{rem}\right)\right]}{E_{abs}-E_{rem}} \end{array}$$

(2) $$\begin{array}{l} N_{rem}=\frac{\left[N_{flo}*\left(E_{abs}-E_{flo}\right)\right]-\left[N_{final}*\left(E_{abs}-E_{final}\right)\right]}{E_{rem}-E_{abs}} \end{array}$$

where N_abs_ (mg organ^−1^) represented the total N absorbed by an organ from anthesis until 2 weeks after anthesis, and N_rem_ was the total N remobilized from sowing until anthesis; N_final_ represented the total N in the organs and E_final_ (%) was the organ ^15^N excess at 2 weeks after anthesis; N_flo_ and E_flo_ represented, respectively, the total N in the organs and their ^15^N excess at flowering; E_rem_ is the ^15^N excess of N absorbed before anthesis and remobilized after anthesis, which was considered to be equal to the ^15^N excess at flowering of the ears and flag leaves combined, and E_abs_ is the ^15^N excess of N absorbed after anthesis from the substrate, which was considered to correspond to the atom% ^15^N of the substrate water solution.

For the mature grains, accumulated N originating from N_abs_ was calculated following Robinson (2001):

(3) N15tracer=(δsample-δbackground)/(δtracer-δbackground)

(4) Ntracer=Nsample*N15tracer

Assuming that the N_abs_ of the grain was represented using the equations resolved from Equations (3) and (4), and that N_rem_ was considered as the N remainder in the grain, we obtained:

(5) $${\text{N}}_{\text{abs}}={\text{N}}_{\text{grain}}*{\text{E}}_{\text{grain}}{\text{/E}}_{\text{abs}}$$

(6) $${\text{N}}_{\text{rem}}={\text{N}}_{\text{grain}}-{\text{N}}_{\text{abs}}$$

Where N_grain_ (mg ear^−2^) was the total grain N content of a whole ear for each genotype; N_abs_ was the N derived from absorption during the reproductive stage (from anthesis to maturity) and N_rem_ was the N derived from remobilization during the vegetative stage (from sowing to anthesis); E_grain_ was equal to labeled plant grain δ^15^N minus non-labeled plant grain δ^15^N from the same plot; E_abs_ was considered to correspond to the δ^15^N of the substrate water solution; and the grain N_abs_ of one ear for each genotype was calculated by using Equation (5). Since N labeling has no effect on N remobilization capacity, and grain N is only derived from N_abs_ and N_rem_, then the remainder of total N and N_abs_ of a complete ear could be considered as the grain N remobilized from the vegetative stages by using Equation (6).

In the current experiment, remobilization efficiency of the N taken up before anthesis (NRE) was calculated by the proportion of N remobilized into (and/or out of) the ear and flag leaf during the 2 weeks after anthesis with the total N in the organs at anthesis. N absorption efficiency after anthesis (NAE) was calculated based on the proportion of N absorbed in the ear and flag leaf during 14 days with the N accumulated at anthesis. Moreover, the grain NRE was calculated based on the proportion of N remobilized at anthesis with the total N in the mature grain. The grain NAE was calculated as the ratio of N absorbed and translocated after anthesis to the grain N absorbed after anthesis.

### Ear and flag leaf rubisco quantification

Aliquots of 1.5 g of frozen flag leaf and 2.5 g of frozen ear (with the growing grains removed) from each plot at both anthesis and 2 weeks after anthesis were ground in a cold mortar with liquid nitrogen. All the following steps were carried out on ice. Ten milliliter of 0.1 M sodium phosphate buffer (pH 7.5) was added to the frozen sample powder and stirred over a period of 15–20 min. The extract was filtered through 4 layers of muslin into a small cold beaker and the filtrates were precipitated with 30% ammonium sulfate prior to centrifugation at 12,000 rpm for 10 min at 4°C. Rubisco concentration measurements were carried out according to the Bradford assay (Bio-Rad) and SDS-PAGE (12.5% polyacrylamide). Gel images were scanned and analyzed using the Typhoon™ Trio Imager (GE Healthcare) densitometer.

### Statistical analyses

A hypothesis of zero difference between means was tested with one-way ANOVA to analyze the genotype effect on the agronomical traits, N_abs_ and N_rem_, of the organs. When the ANOVA was proved significant for any parameter, Tukey's honest significant difference test was conducted for multiple comparisons at *P* ≤ 0.05. In order to test the effect of the genotypic and sampling time effects for the isotope signatures and traits associated with N assimilation, two-way ANOVA tests were performed for organ dry weight, N content and Chl. content and traits associated with Rubisco protein. When the genotypic effect was significant, a LSD test was performed for multiple comparisons at *P* ≤ 0.05. A set of linear correlation procedures was constructed to analyze the relationships between the measured traits using the value of each plot. All the data were analyzed using the SPSS v.20 statistical package (SPSS Inc., Chicago, IL, United States). All the figures were created using the Sigma-plot version 12.0 program for Windows (Systat Software, Point Richmond, CA, United States).

## Results

### Evaluation of yield components and grain N stores

In this experiment, the grain yields per plant of the genotypes Zhoumai 18 and Yumai 20 were higher than genotypes Lankao 198 and Yumai 2 (Table 1). Even though the ear number per plant, and the grain number and grain weight per ear were not significantly changed among genotypes, all these yield components had a similar changing trend with grain yield. The plant height was higher in high yielding genotypes and changed significantly among the genotypes. Moreover, no differences were found in grain dry matter N concentration, but the total N of the mature grains changed significantly and showed the same pattern as grain yield. The grain yield was highly and positively associated with grain number (*r* = 0.80, *P* ≤ 0.01; Table S1). A negative correlation was found between TGW and grain number (*r* = −0.65, *P* ≤ 0.05).

**Table 1:** The mean, standard error of the mean (SEM) and the *P*-value for the ONE-WAY ANOVA test for grain yield per plant (GY) and grain weight per ear (GW), plant height, thousand grain weight (TGW), Ear number per plant (ENP), grain number per plant (GNP), grain nitrogen concentration and content for four Chinese winter wheat genotypes.

	**GY**	**GW**	**Height**	**TGW**	**ENP**	**GNP**	**Grain N concentration**	**Grain N content**
	**(g plant^−1^)**	**(g ear^−1^)**	**(cm)**	**(g)**	**(#)**	**(#)**	**(%)**	**(mg ear^−1^)**
Yumai 2	4.17b	2.27	69.35b	46.88	1.8	98.37	2.20	91.18c
Lankao 198	4.28b	2.13	69.76b	53.16	2.0	87.24	2.49	107.28bc
Yumai 20	5.19ab	2.46	82.87a	43.45	2.1	124.45	2.65	137.61ab
Zhoumai 18	6.23a	2.83	80.51a	47.27	2.2	126.27	2.52	155.66a
Mean	4.97	2.42	75.62	47.69	2.05	109.08	2.47	122.93
SEM	0.31	0.11	2.06	1.48	0.08	6.89	0.07	8.54
*P*-value	0.025	0.107	0.003	0.115	0.608	0.090	0.109	0.040

*Each value is the mean of 3 replicates. For each genotype, mean values followed by the same letter were not significantly different to the Tukey's honestly significant difference test (P < 0.05)*.

### Nitrogen and rubisco concentration of organs at anthesis and grain filling

**Figure 1:**
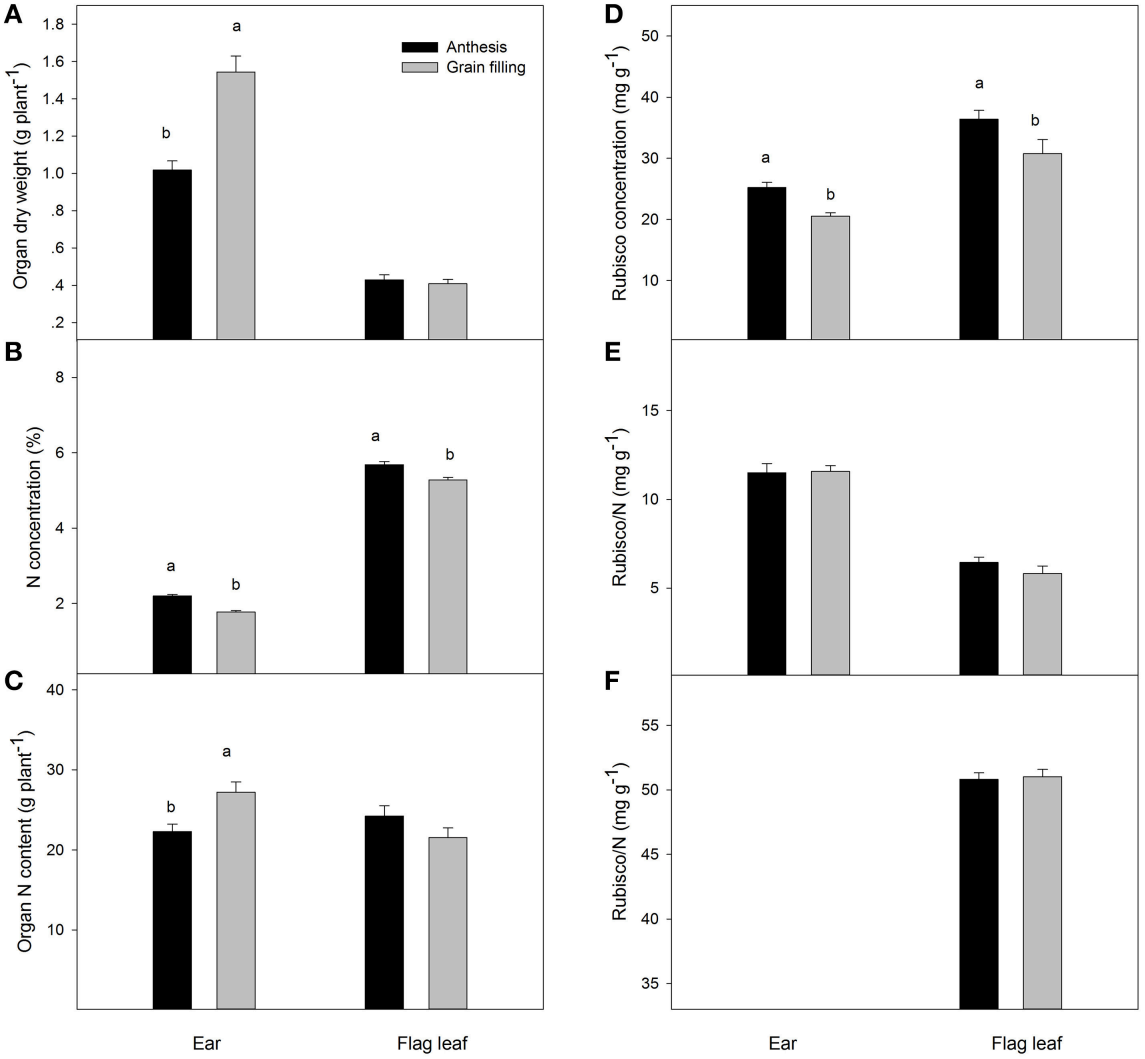
Organ dry weight **(A)**, N concentration **(B)**, whole organ N content **(C)**, Rubisco concentration **(D)**, Rubisco per N **(E)** and Chl. readings (SPAD 520 value) **(F)** of whole ears and flag leaves during anthesis and 2 weeks after anthesis. Mean values with different superscripted letters are significantly different according to the Tukey's honestly significant difference test (*P* < 0.05).

**Figure 2:**
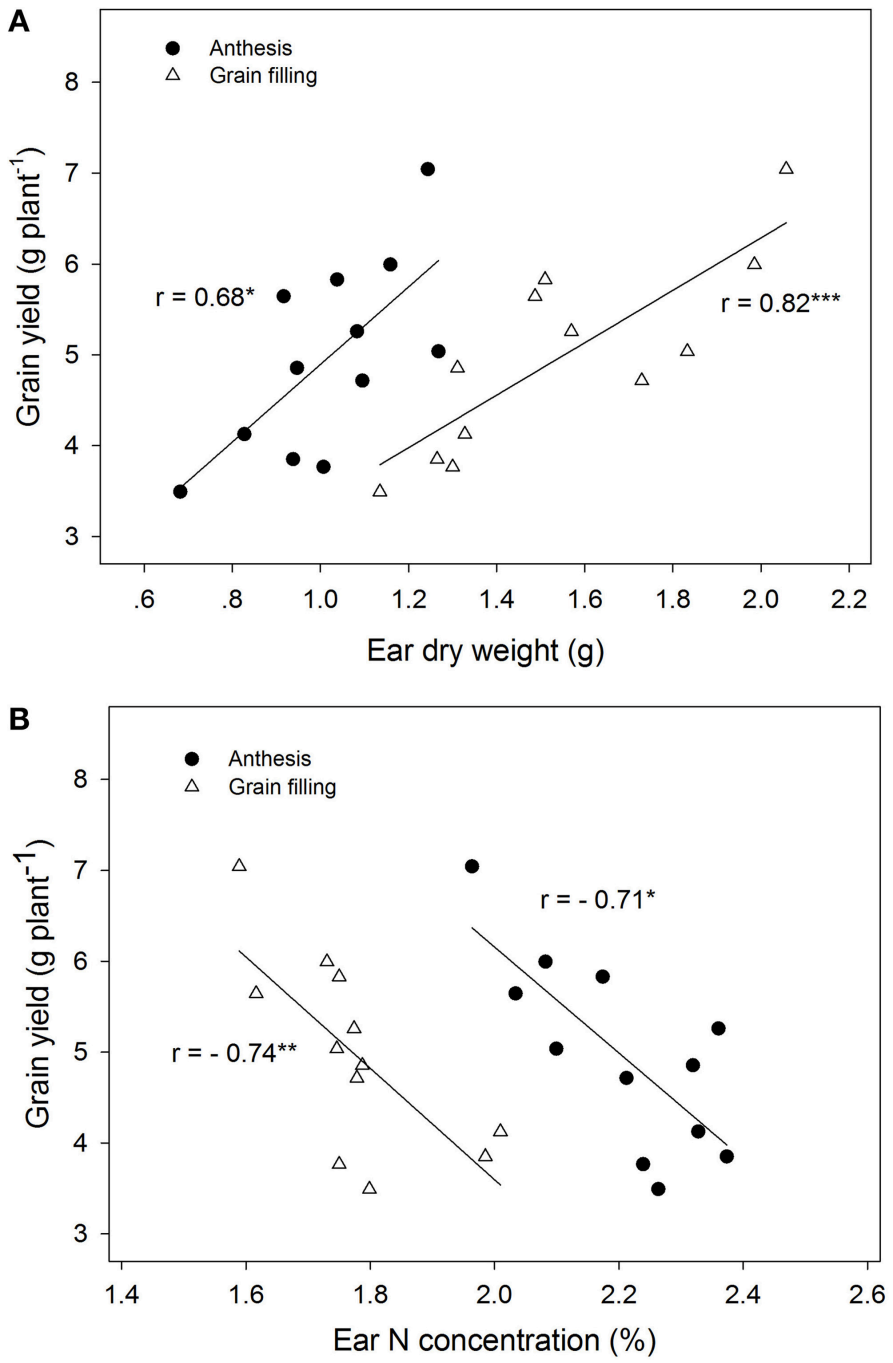
Linear correlations between grain weight and ear dry weight of each plant **(A)** and ear nitrogen concentration **(B)** during anthesis and grain filling for four Chinese winter wheat genotypes. (^*^P < 0.05; ^**^P < 0.01; ^***^P < 0.001).

The ear dry weight at grain filling was significantly higher than at anthesis, and this parameter was also significantly different between genotypes (Figure 1, Table 2). It is interesting to note that the N concentration of the ear changed significantly among genotypes, decreasing from 2.20% at anthesis to 1.78% at grain filling. In contrast, although the dry weight of flag leaves did not change between sampling times, it changed significantly among genotypes and decreased from anthesis to grain filling. The nitrogen content of the whole ear per plant increased from anthesis to grain filling, but the total nitrogen content of flag leaves per plant did not show any differences between sampling times. The grain yield was positively correlated with ear dry weight per plant during both growth stages (*r* = 0.68, *P* ≤ 0.05; *r* = 0.82, *P* ≤ 0.001, respectively), but in contrast the N concentration in the ears was negatively correlated with grain yield during the same periods (*r* = −0.71, *P* ≤ 0.05; *r* = −0.74, *P* ≤ 0.001, respectively) (Figure 2). The same relationships were not found for flag leaf dry weight or N concentration.

**Table 2:** The probability values (P) of a genotype effect (G), sampling time effect (T) and GxT interactions for ear and flag leaf blade dry weight, N concentration, N content per entire organ, Rubisco concentration expressed in dry matter units, Rubisco concentration expressed as total N units and flag leaf Chlorophyll readings (SPAD readings) during anthesis and grain filling for four Chinese winter wheat genotypes grown in pots.

**Parameters**	**Organs**	***G***	***T***	**G × T**
Organ dry weight (g plant^−1^)	Ear	0.004	≤ 0.001	0.366
	Flag leaf	0.025	0.516	0.847
N concentration (%)	Ear	≤ 0.001	≤ 0.001	0.327
	Flag leaf	0.030	≤ 0.001	0.115
Organ N content (mg plant^−1^)	Ear	0.090	0.004	0.597
	Flag leaf	0.045	0.115	0.970
Rubisco concentration (mg g^−1^ DW)	Ear	0.002	≤ 0.001	0.037
	Flag leaf	0.100	0.038	0.139
Rubisco/N (mg g^−1^)	Ear	0.050	0.891	0.025
	Flag leaf	0.125	0.198	0.098
Chl. readings (SPAD readings)	Flag leaf	0.129	0.768	0.999

**Figure 3:**
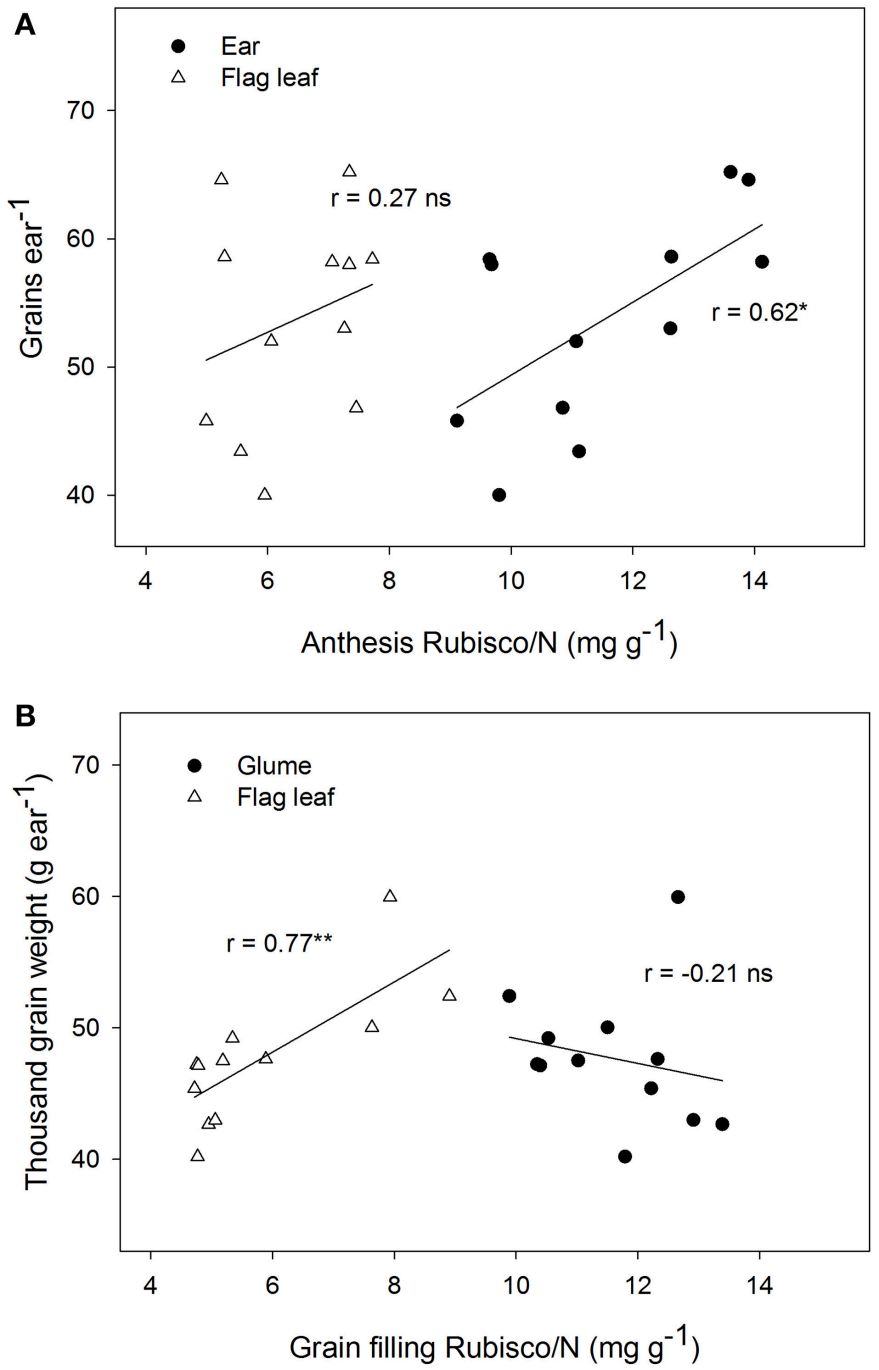
Linear correlations of grain number per ear (grains ear^−1^) with Rubisco content per unit of total nitrogen in the flag leaves and ears at anthesis **(A)**, and thousand grain weight (TGW) with Rubisco content per unit of total nitrogen in the leaves and ears during grain filling **(B)** for four Chinese winter wheat genotypes. (^*^P < 0.05; ^**^P < 0.01; ns, not significant).

The Rubisco concentration of the ear, expressed in dry matter weight units, changed significantly among genotypes and showed lower values than in the flag leaves at both sampling times (Table 3). The Rubisco concentration of the ear, when expressed as N concentration units, increased nearly two-fold in the flag leaves during both growing stages. At anthesis, a positive linear correlation was found between grain number per ear and ear Rubisco/N (*r* = 0.62, *P* ≤ 0.05), but this was not the case in the flag leaves (Figure 3). However, at grain filling, the thousand-grain weight was highly and positively associated with Rubisco/N in flag leaves (*r* = 0.77, *P* ≤ 0.01) but not associated with the ear. It should be noted that the lack of linear relationships may be due to the high variability and the presence of one influential point. The flag leaf chlorophyll concentrations measured with a SPAD-520 did not vary significantly between sampling times and among genotypes, but it correlated positively with ear Rubisco/N at grain filling (*r* = 0.58, *P* ≤ 0.05; Table S2).

**Table 3:** The mean, standard error of the mean (SEM) and the *P*-value for the ONE-WAY ANOVA test for the whole ear and flag leaf blade nitrogen absorption (N_abs_) from anthesis to 2 weeks after anthesis, and nitrogen remobilization (N_rem_) during the same period in four Chinese winter wheat genotypes.

	**N**_**abs**_ **(mg/plant organs)**			**N**_**rem**_ **(mg/plant organs)**		
	**Grain**	**Ear**	**Flag leaf**	**Grain**	**Ear**	**Flag leaf**
Yumai 2	28.11b	1.00	1.01	63.07b	1.48	−3.99
Lankao 198	33.78b	0.84	0.74	73.49b	3.07	−2.86
Yumai 20	50.26a	1.17	1.25	87.35ab	4.08	−5.10
Zhoumai 18	54.14a	1.08	0.90	101.52a	6.86	−2.72
Mean	41.57	1.02	0.97		3.87	−3.67
SEM	3.74	0.09	0.08	5.03	0.81	0.70
*P*-value	0.006	0.691	0.161	0.008	0.092	0.664

*Grain N_rem_ represents the N remobilized from sowing to anthesis in the whole plant, and grain N_abs_ represents the N absorbed from anthesis to maturity and accumulated in the grain. Each value is the mean of 3 replicates. For each genotype, mean values followed by the same letter were not significantly different to the Tukey's honestly significant difference test (P < 0.05)*.

### Estimations of organ N absorption post-anthesis and remobilization pre anthesis

**Figure 4:**
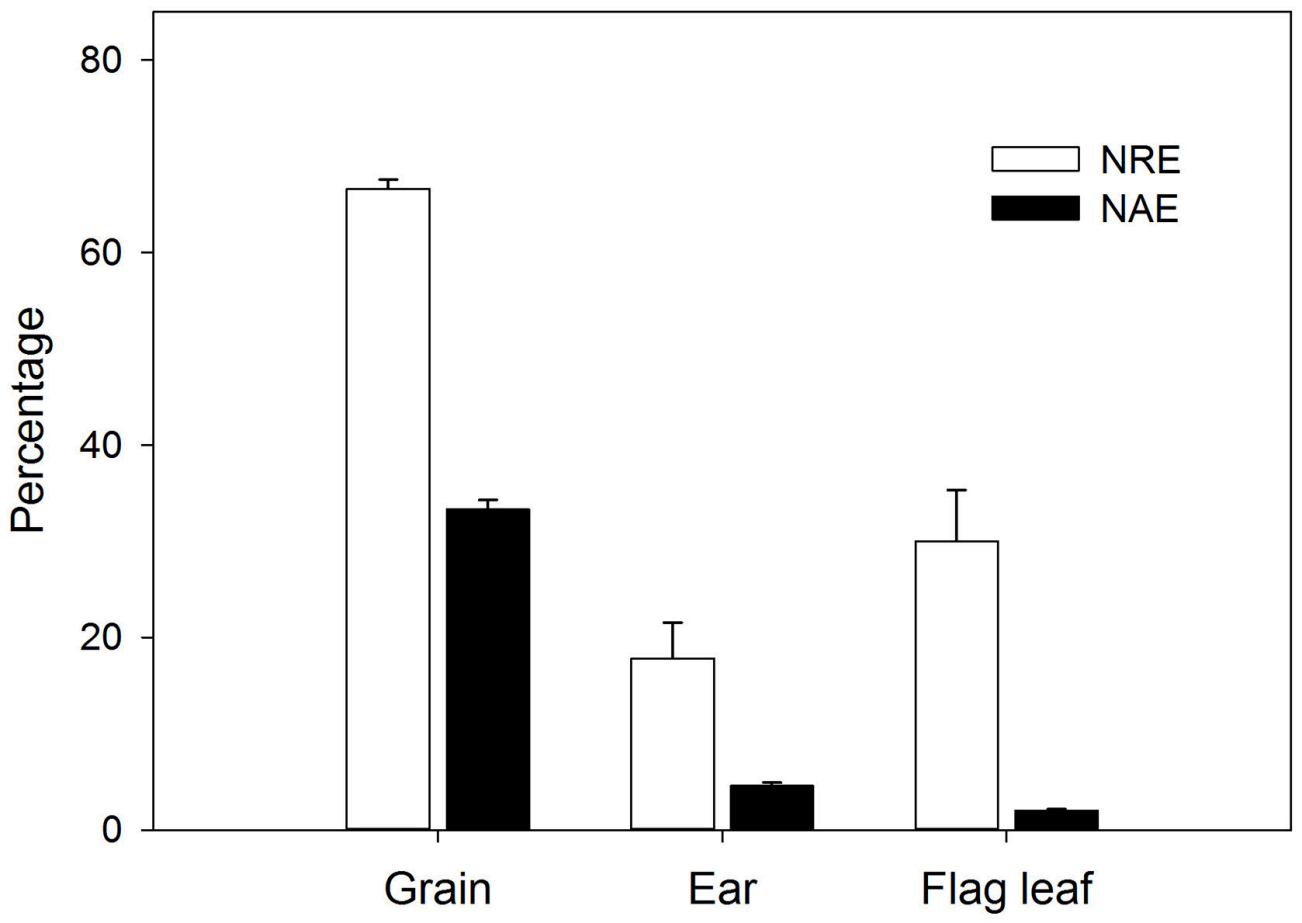
N remobilization efficiency (NRE) and N absorption efficiency (NAE) at 2 weeks after anthesis of the grain, ears and flag leaves. Values represent means across all the genotypes for 3 replicates. Errors bars represent SEM.

The initial plant grain nitrogen absorbed (N_abs_) from root uptake during the whole post-anthesis stage (from anthesis to maturity) and the initial plant grain nitrogen remobilized (N_rem_) from pre-anthesis (sowing to anthesis) showed significant genotypic differences and changes with grain yield (Table 3). During the whole grain filling period, 41.57 mg of N had been absorbed and accumulated in the grains, whereas 81.36 mg of the N was derived from remobilization of pre-anthesis stores. Therefore, the N_rem_ was the main N source that contributed to grain filling, even when growth occurred under optimal conditions. In the ears, the quantity of N_abs_ was 1.02 mg during the 14 days after anthesis, while the N_rem_ was 3.87 mg, indicating that the main source of N was from remobilization. The trend in N_rem_ was for it to increase in the higher-yielding genotypes, but the N_abs_ did not increase in the same way. It is interesting to note that the N_abs_ of the flag leaves was positive (0.97 mg), whereas the N_rem_ of these organs had a negative value (−3.67 mg), which means that a large amount of pre-anthesis N in the flag leaves (compared with ear) had been transferred to other parts of the plant. Therefore, the wheat genotypes in this experiment had achieved a 66.7% N remobilization efficiency (NRE) for the grain throughout the grain filling period, while ears and leaves exhibited 17.8 and 30.0% NRE, respectively, during the 2 weeks post-anthesis (Figure 4). The N absorption efficiency (NAE) of grains, ears and flag leaves was lower than the NRE.

### Relationships between agronomical and physiological traits

Grain yield and grain number per plant were significantly associated with the N of grains derived from N_rem_ (*r* = 0.92, *P* ≤ 0.001; *r* = 0.73, *P* ≤ 0.01, respectively) and N_abs_(*r* = 0.84, *P* ≤ 0.001; *r* = 0.67, *P* ≤ 0.05, respectively; Table 4). The N_abs_ of grain was positively correlated with the N concentration (*r* = 0.59, *P* ≤ 0.05), while the N_rem_ of grain was significantly and positively affected by the ear dry weight at anthesis and grain filling (*r* = 0.63, *P* ≤ 0.05; *r* = 0.82, *P* ≤ 0.001, respectively). The ear N_rem_ was negatively associated with the N concentration (*r* = −0.60, *P* ≤ 0.05) and positively correlated to the ear dry matter Rubisco concentration at anthesis (*r* = 0.73, *P* ≤ 0.01). In contrast, the relationship between ear N_rem_ and the ear dry matter Rubisco concentration changed to negative during grain filling (*r* = −0.63, *P* ≤ 0.05). Although the flag leaf N_rem_ was not correlated with any parameter during either of the growth stages, the flag leaf N_abs_ was negatively affected by the thousand-grain weight (*r* = −0.69, *P* ≤ 0.05) and positively correlated with flag leaf dry weight during both growth stages (*r* = 0.83, *P* ≤ 0.001; *r* = 0.82, *P* ≤ 0.001, respectively).

**Table 4:** Pearson correlation coefficients between the N_abs_, and N_rem_ of the ear, the flag leaf and the grain associated with dry weight, N concentration and Rubisco concentration in the dry matter of the ear (glumes) and the flag leaf blade at anthesis and grain filling, as well as the grain weight, grain number per plant, thousand grain weight (TGW) and grain N concentration at maturity.

		**Ear N_abs_**	**Ear N_rem_**	**Flag leaf N_abs_**	**Flag leaf N_rem_**	**Grain N_abs_**	**Grain N_rem_**
Anthesis	Ear dry weight	0.58	0.22	0.70^*^	−0.16	0.64^*^	0.63^*^
	Ear N concentration	−0.13	−0.60^*^	−0.20	0.10	−0.74^**^	−0.73^**^
	Ear Rubisco concentration	0.50	0.73^**^	0.34	0.51	0.31	0.56
	Flag leaf dry weight	0.49	−0.12	0.83^**^	−0.38	0.28	0.18
	Flag leaf N concentration	−0.55	0.15	−0.69^*^	−0.06	−0.11	−0.17
	Flag leaf Rubisco concentration	0.20	0.28	0.44	0.22	0.10	0.15
Grain filling	Ear dry weight	0.67^*^	0.62^*^	0.62^*^	0.19	0.73^**^	0.82^***^
	Ear N concentration	−0.12	−0.17	−0.27	0.20	−0.59^*^	−0.59^*^
	Ear Rubisco concentration	0.21	−0.63^*^	0.52	−0.18	−0.43	−0.45
	Flag leaf dry weight	0.69^*^	0.27	0.82^***^	0.01	0.38	0.38
	Flag leaf N concentration	0.18	−0.12	0.17	0.21	−0.33	−0.20
	Flag leaf Rubisco concentration	−0.30	−0.13	−0.36	0.34	−0.53	−0.38
Maturity	Grain yield per plant	0.36	0.47	0.29	0.17	0.84^***^	0.92^***^
	Grain number per plant	0.66^*^	0.36	0.66^*^	0.10	0.67^*^	0.73^**^
	TGW	−0.48	−0.20	−0.69^*^	0.16	−0.13	−0.08
	Grain N concentration	0.23	0.42	0.05	0.01	0.59^*^	0.52
N_abs_ & N_rem_	Ear N_abs_	1	0.39	0.73^**^	0.50	0.35	0.43
	Ear N_rem_	–	1	0.10	0.45	0.46	0.63^*^
	Flag leaf N_abs_	–	–	1	0.59^*^	0.20	0.28
	Flag leaf N_rem_	–	–	–	1	−0.03	0.28
	Grain N_abs_	–	–	–	–	1	0.90^***^
	Grain N_rem_	–	–	–	–	–	1

* *P ≤ 0.05*,

** P ≤ 0.01, and

*** *P ≤ 0.001, N = 15*.

## Discussion

### Grain yield determination by grain number and grain weight

In the current study, a high grain yield was obtained compared to the results reported in other trials performed in China (Zheng et al., 2011; Xiao et al., 2012) and Spain (Zhou et al., 2015). The genotypes with higher grain yield exhibited taller shoots and larger grain numbers. The higher yielding genotypes (Zhoumai 18 and Yumai 20) also revealed a larger grain N storage capacity, a better N remobilization capacity from pre-anthesis and a stronger N uptake during post-anthesis that served as an active source to satisfy the high N accumulation capacity of the grains. The taller genotypes Yumai 20 and Zhoumai 18 showed a greater yield formation potential due to the larger grain numbers determined before anthesis, which indicated that the genetic gains in yield in Chinese wheats have been achieved via grain number improvement (Zheng et al., 2011; Xiao et al., 2012). Thus, the negative correlation between TGW and grain number per ear suggested that the total amount of available assimilates was not enough to supply the grain in these genotypes. In that context, the taller genotypes with better yield potential were mainly dependent on their larger storage capacity rather than sourcing N for grain filling. The grain number (53.2 grains per ear), which was higher under our environmental conditions than in the field (31.5 grains per ear) (Xiao et al., 2012), was positively correlated with grain yield, so this may be a hint that selecting genotypes with larger grain numbers can achieve improvements in yield potential during breeding (Zhou et al., 2007; Xiao et al., 2012). This also supports the recent Chinese breeding strategy that is focusing on selecting genotypes with greater ear size and larger grain numbers coupled with a small flag leaf area (in order to improve canopy structure) (Zheng et al., 2011; Zhou et al., 2015). It should be noted that TGW was not associated with grain yield but negatively correlated with grain number (Table S1), however, according to the TGW was dependent on carbohydrate accumulation rather than N assimilate supply, indicating that the total amount of available assimilates was not sufficient for Chinese wheat grain filling (Xiao et al., 2012).

### N assimilation affected by remobilization and uptake

The grain N content is conditioned by both the remobilization of the N assimilated before anthesis and by the N absorbed during the grain filling process (Andersson et al., 2005; Kichey et al., 2007; Masclaux-Daubresse et al., 2008). In the current work, the results of determining whether the N_rem_ or the N_abs_ contributed more to grain filling revealed that 66.7 percent of the N in mature grain was derived from remobilization, which is a lower proportion than most of the cases reported from other parts of the world, such as Europe (71.2%, in the optimal field of French) (Kichey et al., 2007), including Mediterranean conditions (71.5–90%, in N, P treatments field condition) (Dordas, 2009), and Australia (81.0%, growing in greenhouse conditions) (Palta and Fillery, 1993). The NRE changed depending on genotypes and environments (Gaju et al., 2014). In the current experiment a complete Hoagland solution was applied throughout the growth cycle, there was no cold stress (compared with Henan Province, China) and ample illumination was provided, and this indicates that plants were grown under optimal conditions. Likewise, the high yield and TGW indirectly reflected that the plants did not suffer climatic stress. Previous work conducted in well-irrigated fields produced a similar NRE range as this work (Kichey et al., 2007), and also provides further evidence that our plants were not limited by the growing environment. Many previous papers have demonstrated that the NRE of winter wheat is associated with plant senescence traits (Pask, 2009; Gaju et al., 2014). The mature grain N concentration was positively associated with grain N_abs_ rather than N_rem_, which may be due to N_rem_ dominating the N stream before the plants become senescent. By contrast the N absorbed after flowering is invested by the plant in maintaining leaf greenness for prolonged photosynthetic activity, and is not directed toward grain filling. However, the yield and N content of the grain still seemed to be influenced by N that was derived from remobilization from plant tissues rather than absorbed directly by roots during the grain filling period. Therefore, finding the genotype with the highest NRE would be the strategy to improve the N source/sink balance between the plant and grain (Martre et al., 2003). This also suggests that the NRE of Chinese wheat still has room for improvement via enhanced N use efficiency so that increases in yield potential can be achieved.

### N contribution of the ear and flag leaf to grain filling

It is widely accepted that after heading the ear is responsible for depositing N, while the other plant parts serve to feed this organ (Lopes et al., 2006). In the current work it is notable that the ear (with grains removed) had accumulated 3.87 mg of N by 2 weeks after anthesis from other plant parts, whereas the flag leaf had remobilized 3.67 mg of pre-anthesis N to other plant parts. Although the ear N_rem_ was not significantly different among genotypes, the trend indicated that the genotype with the higher grain yield had remobilized more nitrogen through the ear dry matter. Moreover, the quantities of N in both organs were not significantly correlated with each other (data not shown), indicating that the flag N_rem_ was not directly fed into the ear and/or that the ear N stream was not derived from the flag leaf. In the experiment conducted by Barraclough et al. (2014), the distribution of N in different organs at anthesis was in the following descending order: stem (28%) > ear (23%) > flag leaf (15%) > sheaths (14%), and proportionally more was present in the ear (30%) and less in the flag leaf (8%) under low N conditions. Moreover, the ear had accumulated 89% of total N from other plant parts at maturity, which was much higher than the N in the stem (4%) and flag leaf (2%). In that sense, the ear was a critical pool of N deposition that influenced the N accumulation in the grain. Indeed, in our experiments, the ear dry matter (removed kernel) increased from 1.02 to 1.54 g and the N content changed from 10.89 g to 13.30 g during the transition from anthesis to grain filling, respectively, and the positive correlation between ear N_rem_ and grain N_rem_ indicated that the ear was an important organ for partitioning and allocating N to the grain. In contrast, the flag leaf was more distant from grains, and the lack of a significant association between grain N_rem_ and flag leaf N_rem_ demonstrated that the leaf N stream was not directly translocated to the ear, which was contrary to a number of reports that leaf lamina N remobilization determines genetic variation of grain N concentration (Bertheloot et al., 2008; Gaju et al., 2014).

The N partitioning capacity of ear depends on the number of sinks and their activity, whereas grain number is strongly associated with assimilate availability at flowering (Heitholt et al., 1990; Slafer et al., 1990). Optimizing N partitioning to the grain from biomass is an important breeding strategy to improve yield and quality (Baresel et al., 2008). In the present work, the improved yield potential was closely associated with a larger ear sink size (understood as a larger number of grains). The grain N_rem_ was negatively correlated with the ear N concentration throughout grain filling due to the greater optimization of this sink in the high yielding genotypes during breeding. Moreover, many reports have indicated that higher yielding genotypes could be selected on the basis of a higher N concentration in the plant dry matter, with the assumption that this reflects better photosynthetic capacity (Parry et al., 2011). However, for the recently developed Chinese genotypes, the ear N concentration is negatively associated with grain yield, with the ear being the most crucial organ affected during grain filling (Aranjuelo et al., 2013a; Sanchez-Bragado et al., 2014b, 2017; Zhou et al., 2016).

Evaluating whether the NRE of the flag leaf or the NRE of the ear (normally without a significant genotypic effect) was a greater contributor to grain filling was complex because interpretation of the effect of the NRE on agronomical traits is difficult due to the many mechanisms are involved (Kichey et al., 2007; Pask et al., 2012). For bread wheat growing under optimal conditions, the amount of grain N contributed directly by the flag leaf blade was comparatively low in the 2 weeks after anthesis, even though our methods had the limitation of not taking into account the potential influence of other organs during grain filling, such as the rest of the stem (peduncle, flag leaf sheath, lower leaves). Despite this, the present results support the concept that breeding for yield potential improvement should focus on genotype selection for larger ear size, to form greater numbers of grains and accumulate more N during pre-anthesis. Subsequently, the larger ear total organic matter during post-anthesis is essential to serve as a source of N for remobilization and to modulate the N stream derived from other plant parts during grain filling. Although the N concentration of the ear may be diluted (presented in lower N concentration) before anthesis in the ear of high-yielding genotypes, adequate N remobilization in the ear during post-anthesis, which is derived from higher total N accumulation (in terms of larger biomass), needs to be considered during breeding strategies.

### N contribution to grain filling: total organic matter and rubisco

A decreasing trend in N concentration in most plant parts is the natural process during grain filling in wheat, and in many cases it varies greatly with environmental factors and can also be affected by genotype (Le Gouis et al., 2000; Pang et al., 2014). High yielding genotypes exhibit lower N concentrations in the ear, and this N dilution effect may be due to an interaction between the expansion of dry matter in the ear and N feeding to satisfy the sink demand (Demotes-Mainard et al., 1999; Zhou et al., 2014). At the same time, in our research, although the dry weight of the flag leaf was not changed between growth stages, the decreasing N concentration indicates the N movement (move out) caused by N remobilization, but this N movement was not due to plant senescence (measured as no change between Chl readings in the flag leaves between sampling times). In normal breeding strategies, selecting optimized Rubisco function and regulation genotype is linked to improved plant resource use efficiency (including N and light etc.) (Parry et al., 2011; Lin et al., 2014). Traditionally it has been assumed that N derived from Rubisco in the flag leaf was the main N source to feed filling grains (Bertheloot et al., 2008), and this was mainly due to Rubisco typically accounting for up to 60 percent of the total soluble protein and 30 percent of the total N (Carmo-Silva and Salvucci, 2013; Carmo-Silva et al., 2015; Zhou et al., 2016). In this experiment, the percentage of N allocated to Rubisco (Rubisco/N) in the ear was nearly twice that of the flag leaf, which indicated that the ear accumulated extra Rubisco, and that this apparent over-investment in Rubisco is likely to provide a means of storing N (Makino, 2011). Although the way that Rubisco-derived N distributed among the organs affects grain filling is not fully understood, the literature suggests that Rubisco-derived N from the ears plays a significant role grain filling through amino acids released from protein degradation of Zhou et al. (2016). In the current experiment, a positive correlation between the amount of N_rem_ and the Rubisco concentration in the ear at anthesis was observed, with the correlation becoming negative after this time, confirming the importance of Rubisco regulation in the ear, which is closely associated with N remobilization (Carmo-Silva et al., 2015).

In conclusion, N is one of the most important nutrients needed for wheat growth and grain formation. The high-yielding Chinese wheat genotypes were characterized by larger grain number and larger ear sizes. Grain filling was mainly supplied with N remobilized from pre-anthesis biomass accumulation, the ear being a critical organ for balancing sink strength and N source activity. Rubisco in the ear during pre-anthesis acted as an N reservoir and served as an N source during grain filling. For modern Chinese wheat breeding, the recommended strategy is to thus focus on genotypes with higher Rubisco concentrations in the ear before anthesis. This germplasm could significantly improve N absorption, and its remobilization during pre-anthesis contributes to grain filling, resulting in higher grain N accumulation and better yield performance.

## Author contributions

BZ performed the greenhouse experiments. SS and MS conducted the analyses in the laboratory and JP and ZL supervised the study. All authors contributed to writing the manuscript.

### Conflict of interest statement

The authors declare that the research was conducted in the absence of any commercial or financial relationships that could be construed as a potential conflict of interest.
