# Supplementary material for: Relative Contribution of Nitrogen Absorption, Remobilization, and Partitioning to the Ear During Grain Filling in Chinese Winter Wheat
Source: Front Plant Sci. 2018 Sep 19;9:1351. doi: 10.3389/fpls.2018.01351 (PMC6156426; doi:10.3389/fpls.2018.01351)
Supplement: Supplementary file 1 [file Table_1.PDF]

Table S1: Pearson correlation coefficients of the relationship between grain weight per plant, nitrogen concentration (N%), thousand grain weight, grain N concentration at maturity, and ear dry weight, ear N%, Rubisco concentration, and flag leaf chlorophyll content (Chl; SPAD units), dry weight, N%, Rubisco concentration at anthesis for four wheat genotypes. * P≤0.05; **P≤0.01; and ***P≤0.001.

Anthesis stage		Grain				Ear				Flag leaf		
		Weight per plant	Grains per plant	TGW	N %	Dry weight per plant	N%	Rubisco%	Rubisco/N	Chl	Dry weight per plant	N%
Grain	Grains per plant	0.80**										
	TGW	-0.21	-0.65*									
	N %	0.18	0.14	0.16								
Ear	Dry weight per plant	0.68*	0.81**	-0.56	0.20							
	N%	-0.74**	-0.52	0.28	-0.30	-0.54						
	Rubisco%	0.35	0.37	-0.18	0.45	0.31	-0.43					
Flag leaf	Chl	0.21	0.42	-0.4	0.06	0.11	-0.23	-0.04	0.05			
	Dry weight per plant	0.28	0.68*	-0.79**	0.02	0.802**	-0.17	0.06	0.12	0.23		
	N %	-0.19	-0.63*	0.51	-0.02	-0.41	-0.23	-0.17	-0.03	-0.34	-0.69*	
	Rubisco %	0.19	0.25	-0.29	0.01	0.15	-0.02	0.01	0.02	-0.18	0.31	-0.32
