# Supplementary material for: Relative Contribution of Nitrogen Absorption, Remobilization, and Partitioning to the Ear During Grain Filling in Chinese Winter Wheat
Source: Front Plant Sci. 2018 Sep 19;9:1351. doi: 10.3389/fpls.2018.01351 (PMC6156426; doi:10.3389/fpls.2018.01351)
Supplement: Supplementary file 2 [file Table_2.PDF]

Table S2: Pearson correlation coefficients of the relationship between grain weight per plant, nitrogen concentration (N%), thousand grain weight, grain N concentration at maturity, and ear dry weight, ear N%, Rubisco concentration, and flag leaf chlorophyll content (Chl; SPAD units), dry weight, N%, Rubisco concentration at grain filling for four wheat genotypes. * P≤0.05; **P≤0.01; and ***P≤0.001.

Grain filling stage		Grain				Ear			Flag		
		Weight plant^{-1}	Grains plant-1	TGW	N %	Dry weight per plant	N%	Rubisco/N	Chl	Dry weight	N%
Grain	Grains per plant	0.80**									
	TGW	-0.21	-0.65*								
	N %	0.18	0.14	0.16							
Ear	Dry weight per plant	0.82**	0.86**	-0.54	0.29						
	N%	-0.71*	-0.54	0.22	-0.01	-0.57					
	Rubisco%	-0.40	-0.12	-0.07	-0.24	-0.19	0.27				
Flag leaf	Chl	0.17	0.51	-0.45	0.09	0.40	-0.53	0.58*			
	Dry weight per plant	0.38	0.70*	-0.76**	-0.01	0.71*	-0.07	0.40	0.31		
	N%	-0.08	0.04	-0.14	-0.49	-0.09	-0.38	0.30	0.60*	-0.11	
	Rubisco%	-0.52	-0.71*	0.72**	0.00	-0.65*	0.39	0.15	-0.15	-0.59*	0.26
